# Tunable gold nanoparticle synthesis using microfluidic flow focusing enabled by reusable 3D-printed multimaterial connectors

**DOI:** 10.1007/s00604-026-07848-4

**Published:** 2026-02-19

**Authors:** Muhammad Mubashar Saeed, Bilal Javed, Eadaoin Carthy, Nicholas Dunne, David Kinahan

**Affiliations:** 1 ML-Labs Centre for Research Training, Dublin City University, Dublin, Ireland; 2 School of Mechanical and Manufacturing Engineering, Dublin City University, Dublin, Ireland; 3 Biodesign Europe, Dublin City University, Dublin, Ireland; 4 RAPID Institute, Dublin City University, Dublin, Ireland; 5 School of Food Science and Environment Health, Technological University Dublin, Dublin, Ireland; 6 Nanolab Research Centre, Physical to Life Sciences Research Hub, Technological University Dublin, Dublin, Ireland; 7 Centre for Medical Engineering Research, School of Mechanical and Manufacturing Engineering Dublin City University, Dublin City University, Dublin, Ireland; 8 Advanced Manufacturing Research Centre (I-Form), School of Mechanical and Manufacturing Engineering, Dublin City University, Dublin, Ireland; 9 SFI Research Centre for Medical Devices (CÚRAM), University of Galway, Galway, Ireland; 10 Advanced Materials and Bioengineering Research Centre (AMBER), Trinity College Dublin, Dublin, Ireland; 11 School of Pharmacy, McClay Research Centre, Medical Biology Centre, Queen’s University Belfast, Belfast, UK; 12 Department of Mechanical and Manufacturing Engineering, School of Engineering, Trinity College Dublin, Dublin, Ireland; 13 Trinity Centre for Biomedical Engineering, Trinity Biomedical Sciences Institute, Trinity College Dublin, Dublin, Ireland

**Keywords:** Gold nanoparticles synthesis, Microfluidics, World to chip, 3D printed microfluidics, Microfluidic connectors, Catalytic activity

## Abstract

The design and fabrication of reusable, cost-effective, and leakage-resistant microfluidic connectors using a commercial multi-material 3D printer (Connex Objet 3D, Tri-Tech 3D, UK) is reported. The connectors, comprising plugs, adapters, and ports, were fabricated from VeroClear™ (rigid polymer) and incorporated TangoBlack (elastomeric soft polymer) as integrated ferrules and gaskets. This multi-material approach enabled the attachment of the connectors using physical locking mechanisms; thus eliminating the need for adhesives and so enabling reusability without mechanical failure. These 3D printed plugs and ports are demonstrated by the synthesis of gold nanoparticles (AuNPs) in a HEPES-mediated microfluidic system under different flow rate ratios (FRR). The resulting AuNPs are evaluated for catalytic performance in the model reduction of 4-nitrophenol to 4-aminophenol. Precise control overflow FRR enabled tunable modulation of AuNPs size, polydispersity, and surface plasmonic responses (SPR). The resulting NPs demonstrated excellent batch-to-batch consistency, critical for reproducible analytical performance. This work presents a scalable interfacing strategy by featuring 3D printed microfluidic connectors, for microfluidic based NPs synthesis platforms and highlights that variation of FRR serves as an effective tool to tailor nanoparticle properties while offering mechanistic insight into AuNP formation dynamics under varying flow conditions.

**Graphical abstract:**

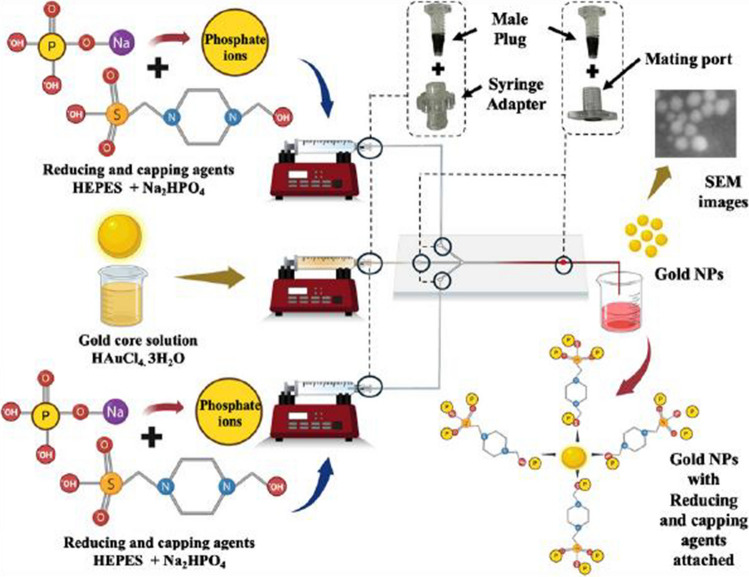

**Supplementary Information:**

The online version contains supplementary material available at 10.1007/s00604-026-07848-4.

## Introduction

Microfluidics is defined by the precise manipulation of fluids within microscale architectures, typically encompassing channel and chamber dimensions that facilitate handling volumes ranging from 10⁻⁹ to 10⁻^1^⁸ L. This technology has demonstrated significant advantages in the synthesis of NPs, addressing critical limitations inherent to conventional batch processes, such as inefficient reagent utilization, prolonged reaction times, and suboptimal control over physicochemical reaction parameters [1]. Microfluidic platforms enable continuous or segmented flow regimes, which support homogeneous reaction environments and facilitate rapid and efficient mixing of reactants, thereby enhancing the reproducibility and uniformity of NP synthesis [2–4].

One of the principal benefits of microfluidic NP synthesis is the capacity for precise modulation of particle size, morphology, and dispersity, attributes that are essential for applications spanning catalysis, biomedicine, and sensing [2, 5]. Precisely engineered microfluidic channels are critical in modulating the reactants’ concentration gradients and flow dynamics, specifically, the gold precursor (HAuCl₄) and the reducing agent (HEPES buffer and Na_2_HPO_4_). By enabling fine control over mixing efficiency and reaction time at the microscale, the microfluidic environment allows for uniform nucleation and growth of AuNPs. This precise control facilitates the reduction of gold ions by HEPES, leading to the consistent formation of gold nuclei with improved reproducibility and size uniformity compared to conventional bulk synthesis methods [6]. Moreover, microfluidic systems are amenable to integration with real-time analytical and feedback control modules, to advance and address the limitations associated with the nanomaterials process optimization, reproducibility and scalability for their biomedical applications.

**Fig. 1:**
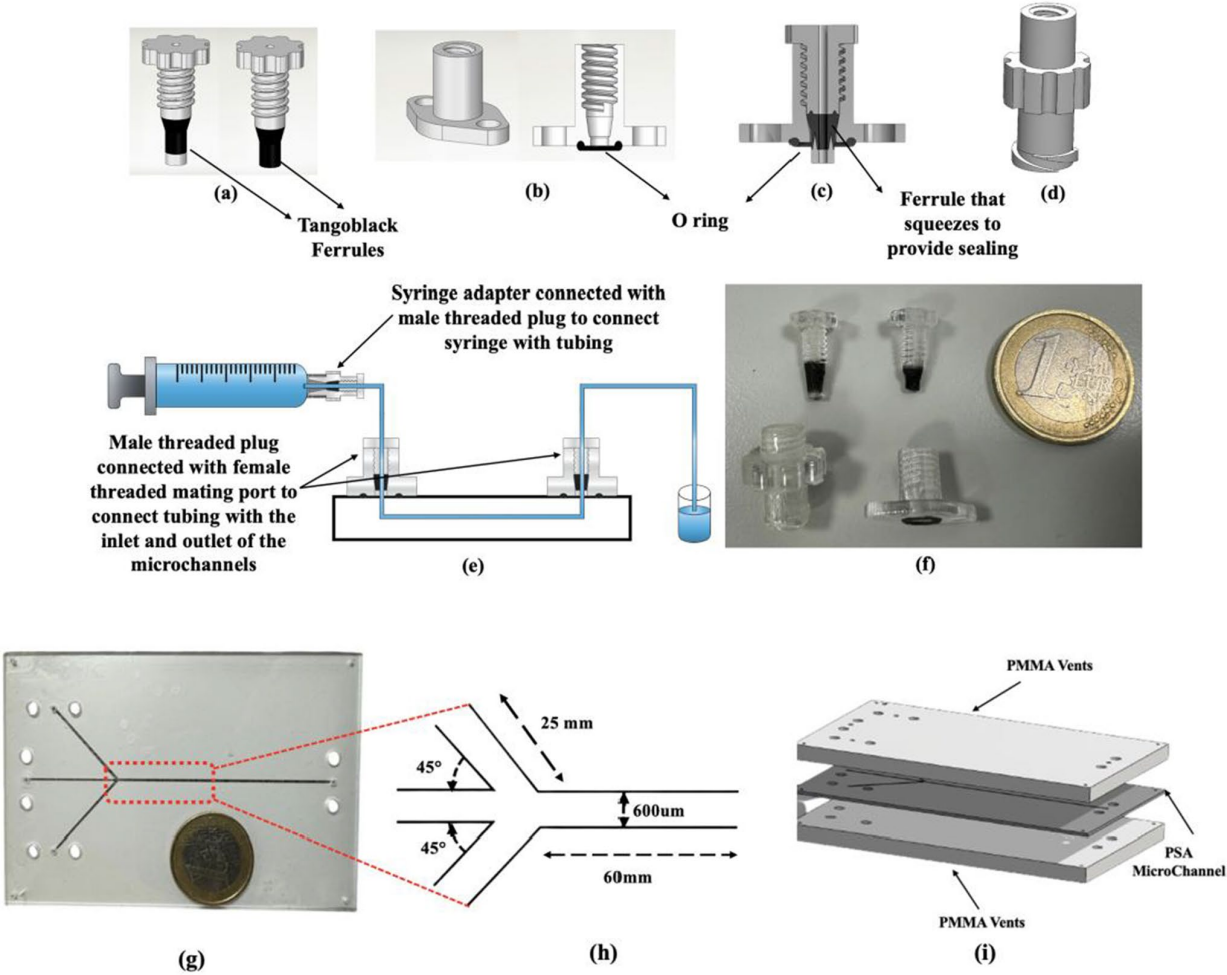
3D-printed multi-material microfluidic connectors. (**a**) Threaded male plug, shown with and without rigid base. (**b**) Corresponding mating port. (**c**) Cross-sectional view of assembled male plug and mating port. (**d**) Syringe adapter. (**e**) Schematic of connector assembly with syringe and microfluidic chip. (**f**) Photograph of assembled connectors adjacent to a one-euro coin for scale. (**g**) Assembled microfluidic chip shown next to a one-euro coin for scale. (**h**) Microchannel dimensions (**i**) Computer-aided design rendering of the microfluidic chip

Despite these advantages, a persistent challenge in microfluidic system integration is the development of reliable, leak-resistant, and user-friendly interfaces between external reagent reservoirs and the microchannel network [7]. Commercially available connectors are often costly, single-use, and lack the flexibility required for custom device geometries, thereby impeding widespread adoption and scalability. Recent research has focused on the design of alternative world-to-chip connector solutions that prioritise cost-effectiveness, reusability, and robust sealing performance. Table 1 summarises important advances in connector technology for microfluidic applications. In this work, we report the design and fabrication of multi-material, 3D-printed connectors, including plugs, adapters, and mating ports, engineered for reusability, cost-efficiency, and leak-free operation. Our approach leverages the integration of soft elastomeric (TangoBlack) and rigid (VeroClear™) photopolymers within a single component (Fig. 1a), enabling the formation of compliant ferrules and gaskets that provide effective sealing without the need for adhesives or additional assembly steps (Fig. 1b). This multi-material configuration offers distinct advantages over traditional single-material connectors, particularly in terms of mechanical robustness and operational longevity.

**Table 1:** Key features of world-to-chip microfluidic connectors for NP synthesis applications

World to chip solutions	Key characteristics	Reference
Magnetic Connector	Exhibit favorable mechanical properties and provide a moderate interconnect density. Characterized by low manufacturing costs, straightforward assembly procedures, and reusability	[16, 17]
Ultrasonic Welding	Yields assemblies with good mechanical integrity and achieves a moderate level of interconnect density. However, its application is occasionally limited by the propensity for channel clogging, particularly when employed with thermoplastic substrates. Additionally, the resulting welded interfaces are irreversible, precluding the reuse of joined components	[18, 19]
Adhesive Glueing	This method exhibits good mechanical properties, supports high interconnect density, and is compatible with a wide range of materials. However, it is susceptible to clogging. The fabrication process is cost-effective and efficient. Notably, the bonded components are not reusable	[20, 21]
Deep reactive ion itching (DRIE) interlock structures	These silicon-based structures exhibit high interconnect density but possess limited mechanical strength. They are inherently resistant to clogging. However, their fabrication is costly and inefficient, and the structures are not amenable to reuse	[22–24]
Monolithic 3D-printed connectors	Exhibit adequate mechanical strength and moderate interconnect density. Fabricated from photosensitive resins or thermoplastics, they demonstrate resistance to clogging during operation. The manufacturing process is efficient and cost-effective. Reusability is contingent upon the specific material used	[25–27]

Following mechanical validation, the connector system was implemented in the microfluidic synthesis of AuNPs, a class of nanomaterials with broad relevance in diagnostics, therapeutics, and biosensing [8–10]. Functionalized AuNPs demonstrate favorable biocompatibility and tunable biodistribution, supporting their use in targeted therapeutic strategies such as precision medicine [11–13]. Additionally, AuNPs have been incorporated into various point-of-care (PoC) assays, offering rapid, cost-effective diagnostics suitable for deployment in resource-limited settings [14, 15].

The synthesis of gold AuNPs proceeds via a two-step mechanism: (1) reduction of Au^3^⁺ or Au⁺ to Au°, followed by (2) introduction of stabilising agents to regulate particle size and inhibit aggregation [32]. Table 2 summarizes different microfluidic methodologies, emphasizing the parameters investigated during AuNP synthesis.

**Table 2:** Summary of recent microfluidic methodologies for AuNP synthesis, and key experimental parameters evaluated

Approach	Parameters studied	Reference
Two Inlet Microfluidic Chip	Different molar ratios of HEPES	[28]
Hydrodynamic Focusing-Based Microfluidic Chip	2D and 3D hydrodynamic flow focusing, as well as different molar concentrations of reductant	[29]
Segmented Flow Microfluidic Chip	Different media (Air, Oil, and Toluene) for segmented flow generation	[30]
3D-Printed Microfluidic Chip	Different reductant concentrations at different flow rates	[31]

In the present study, AuNPs were synthesized using HEPES buffer and disodium phosphate (Na₂HPO₄), which functions as both reducing and capping agents in a disproportionation-based chemical process [33, 34]. The influence of flow rate ratios (FRR) on NP properties was systematically investigated. As there compared to the previous studies which have examined HEPES-mediated reduction of HAuCl₄ using conventional batch methods [35, 36], microfluidic approach presented in this paper enables continuous reagent delivery at precisely controlled flow rates via syringe pumps, affording enhanced control over reaction conditions and improved reproducibility in AuNP synthesis.

The synthesised AuNPs were subsequently used as catalysts for the reduction of 4-nitrophenol to 4-aminophenol in the presence of sodium borohydride (NaBH₄). 4-NP is a toxic environmental pollutant commonly used in producing pesticides, pharmaceuticals, and industrial chemicals. Conventional remediation methods face challenges in efficiency and complexity. Catalytic reduction of 4-NP to 4-AP is a promising alternative, as 4-AP is valuable in pharmaceutical synthesis. AuNPs have shown excellent catalytic performance for this reaction due to their unique surface and electronic properties, with shape and size significantly influencing activity.

## Materials and methods

### 3D printed connectors

For the fabrication of the 3D-printed connectors, VeroClear™ (a transparent rigid photopolymer) and TangoBlack (an elastomeric photopolymer) were selected as the structural and sealing materials, respectively. These materials (Stratasys Tri-Tech 3D, UK) were processed using a Connex260 Object 3D printer (Stratasys Tri-Tech 3D, UK), which adopts PolyJet technology to enable simultaneous deposition of multiple materials within a single build. This capability allows for integrating compliant sealing elements and rigid structural features into a single connector, thereby optimising both mechanical stability and leak resistance.

### 3D Printing of threaded male plug

A reusable, multi-material male threaded plug was fabricated via 3D printing, using VeroClear™ for the threaded body (Fig. 1a). The plug incorporates an integrated soft TangoBlack ferrule, which provides a compressive seal around the inlet tubing upon tightening. The Connex printer enables direct co-printing of VeroClear™ and TangoBlack, eliminating the need for post-fabrication assembly. The TangoBlack ferrule is specifically designed to interface with microchannel inlets and outlets, facilitating efficient and reliable connection of polytetrafluoroethylene (PTFE) tubing. Under applied torque, the ferrule undergoes elastic deformation, ensuring a tight seal around the tubing. Ferrule dimensions were optimised through mechanical testing: insufficient length resulted in inadequate sealing, whereas excessive length led to mechanical failure under shear stress. The computer-aided design (CAD) specifications and average printed thread dimensions are provided in Table 1S. The difference in the CAD design and 3D Printed parts is very negligible which shows the capability of Connex 3D printer.

During performance testing, mechanical failure was observed at the ferrule-tube interface, where sometimes the ferrule could detach after 3–5 use cycles. To improve durability and reusability, the design was modified to incorporate a rigid VeroClear™ base beneath the ferrule. This structural enhancement maintained effective compression of the ferrule around the tubing during tightening, resulting in a robust, watertight seal. The addition of the VeroClear™ base also facilitated repeated opening and closing of the connector, enabling reliable reuse over at least five cycles without compromising sealing performance.

#### 3D Printing of threaded female mating ports

The multi-material threaded mating port was also fabricated using the Stratasys Connex 3D printer, using the same process as for the male connectors (Fig. 1b). The port features a rigid VeroClear™ body integrated with a compliant O-ring composed of TangoBlack. The TangoBlack O-ring is essential for achieving a secure, leak-tight seal when interfacing the mating port with a PMMA-based microfluidic chip. Assembly and disassembly are facilitated by securing the port to the chip with 3 mm nuts, enabling straightforward reuse. This design allows the mating ports to be employed repeatedly until the TangoBlack O-ring exhibits wear, thereby enhancing both the practicality and cost-effectiveness of the system. Additionally, the internal female threads of the mating port are precisely aligned with the male connectors, ensuring a robust and seamless connection (Fig. 1c).

#### 3D Printing of female syringe adapters

To facilitate the connection between syringes and PTFE tubing, 3D-printed syringe adapters were fabricated using VeroClear™ photopolymer on a Stratasys Connex 3D printer. The Luer lock adapter conforms to ISO 594–2:1998, ISO 80369 [37], and DIN/EN 1707:1996 standards, featuring a 6% conical taper with a 3.44° opening angle (1.72° from the cone axis, corresponding to a 3% slope or 0.03 mm/mm) to ensure compatibility with commercially available syringes (Fig. 1d). The internal threading of the adapter matches that of the mating ports, allowing seamless integration with the male threaded plug that secures the PTFE tubing. The TangoBlack ferrule on the male plug provides an effective seal at the interface between the syringe adapter and the plug. Figure 1e shows the complete assembly, with a glass syringe and steel Luer connected to the 3D-printed adapter and male threaded plug holding the PTFE tubing.

#### Post-processing of 3D printed parts: support removal techniques

Complete removal of support material is important to ensure the proper function of 3D-printed microfluidic connectors. Residual support material can be dislodged during operation and transported into microchannels, resulting in blockages and compromised device performance. Accordingly, immediately following fabrication, all printed components were immersed overnight in a 5% NaOH solution to dissolve support residues. The parts were then thoroughly rinsed in a dedicated washing station to remove any remaining contaminants. Representative images of fully cleaned 3D-printed connectors are shown in Fig. 1f.

#### Material for microfluidic chip

For the fabrication of the microfluidic chip, pressure-sensitive adhesive (PSA) (ARCare7080) films and polymethyl methacrylate (PMMA) (Radionics) sheets 1.5 mm thickness for top layer and 0.5 mm thickness for were used. PSA layers were patterned using a Garment Pattern Cutting Plotter CE7000-130AP at RAPID facility DCU, while through-holes in PMMA substrates were fabricated with Epilog laser Zing 16/24 at Nano Research Facility (NRF) DCU. The dimensions of the resulting microchannels were verified using a Keyence optical microscope to ensure fabrication accuracy.

#### Microfluidic chip development

A three-layer microfluidic chip was designed using SolidWorks 2021 (Fig. 1g). The device is comprised of a vent layer, a microchannel layer, and a base layer, with the corresponding CAD model exported in DXF format for fabrication. Microchannels were patterned onto the PSA film, while the vent and base layers were fabricated from 1.5 mm and 0.5 mm thick PMMA sheets, respectively. Assembly was achieved by sandwiching the PSA microchannel layer between the two PMMA layers (Fig. 1g). The chip featured two side microchannels (600 μm width × 86 μm height × 25 mm length), which intersected the central channel at a 45° angle. The central channel extended 60 mm downstream from the junction (Fig. 1h). This multilayer configuration enabled precise channel definition and robust device assembly suitable for microfluidic applications.

### Reliability assessment of microfluidic connectors

#### Investigation of connector leakages under different flow conditions

The microfluidic connectors were evaluated for leakage using dyed water at flow rates ranging from 100 to 6000 μL min⁻1, taking into account the typical total flow rate (TFR) range for NP synthesis. No leakage was observed during ten consecutive continuous operations, each corresponding to the complete drainage of a 5 mL glass syringe. and the connectors maintained integrity after five consecutive reuse cycles under identical conditions. The corresponding pressures at these flow rates were calculated using the Hagen–Poiseuille equation as mentioned in Table 2S.

#### Investigation of connector leakages under different pressure conditions

Microfluidic connectors are commonly characterized by their maximum pressure tolerance, defined as the highest pressure at which they maintain a leak-free seal. In NP synthesis applications, these connectors are integrated into systems where syringe pumps deliver reagents into microchannels for controlled mixing. To accurately replicate the operational conditions encountered during NP synthesis, a custom 3D-printed test apparatus was developed to apply controlled pressure directly to the syringe plunger, thereby simulating the mechanical load experienced during typical system operation (Fig. 1S). Pressure testing was performed using microfluidic devices comprising PSA-based straight channels sandwiched between two PMMA layers. One channel inlet was connected to a syringe, while the opposite end was sealed to establish a dead-end configuration.

#### Investigation on solvent compatibility

In this work 1 and 10 mM aqueous solutions of chemical regents as mentioned in Section "Chemical regents and materials" have been employed for AuNPs synthesis but in order to depict versatility of our 3D Printed connectors these have been tested for compatibility with different type of solvents along with aqueous solution of HEPES and Na_2_HPO_4_. 1 cm^3^ cubes of VeroClear and tangoblack were printed and dipped into these three different solvents. 1. Water, 2. Ethanol, and 3. Acetonitrile. Dimensions and weight of these cubes after regular intervals.

### Gold nanoparticle synthesis

#### Chemical regents and materials

Gold(III) chloride trihydrate (HAuCl₄·3H₂O), N-(2-hydroxyethyl)piperazine-N'-(2-ethanesulfonic acid) (HEPES) buffer, and sodium phosphate dibasic (Na₂HPO₄) were obtained from Sigma-Aldrich (Ireland) and used as received, without further purification. Deionised water (DI) was generated using a PURELAB Flex 2 water purification system and employed in all solution preparations. For the evaluation of the catalytic activity of the synthesised AuNPs, 4-nitrophenol (4-NP) and sodium borohydride (NaBH₄) were purchased from Fisher Scientific. All reagents were of analytical grade and used as supplied.

#### Microfluidic synthesis of gold nanoparticles: system configuration and flow parameter optimisation

**Fig. 2:**
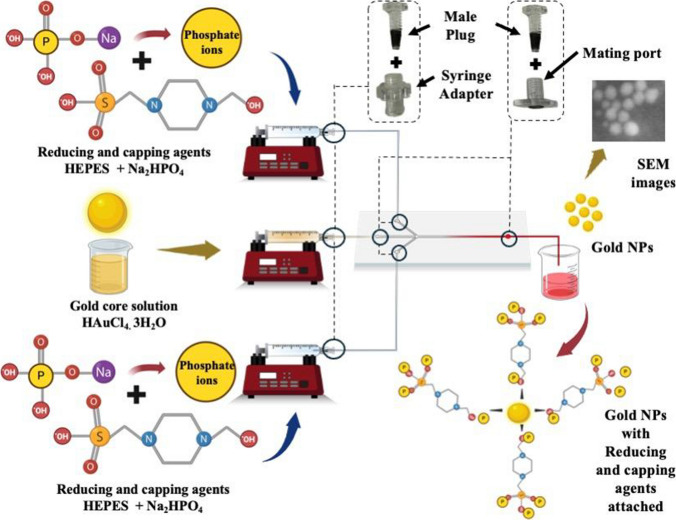
Schematic illustration of the microfluidic-assisted synthesis of AuNPs. The microfluidic setup precisely controls the flow rates and mixing of reagents, including the gold precursor solution (HAuCl₄·3H₂O) and reducing and stabilizing agents (HEPES and Na₂HPO₄). HEPES serves as both a reducing and capping agent, facilitating gold ion reduction through the oxidation of its tertiary amine groups, forming stable gold nuclei. Na₂HPO₄ provides phosphate ions that enhance nanoparticle stabilization and prevent aggregation by electrostatic repulsion. The configuration clearly illustrates microchannels, syringe pumps, and connectors facilitating the precise interaction of reagents in the reaction zone. The resulting AuNPs exhibit controlled size and dispersion, confirmed by SEM imaging. This microfluidic approach enables precise manipulation of reaction kinetics, promoting uniform nucleation and nanoparticle growth with consistent physicochemical characteristics

Stock solutions of HAuCl₄·3H₂O (1 mM), HEPES buffer (10 mM), and Na₂HPO₄ (10 mM) were prepared using DI water. Figure 2 illustrates the schematic of the microfluidic platform employed for the AuNPs synthesis. AuNPs were synthesised via hydrodynamic flow focusing (HFF), wherein an aqueous solution of HAuCl₄·3H₂O was introduced through the central inlet of the microfluidic device, while a premixed solution of HEPES and Na₂HPO₄ (1:3 volumetric ratio) was supplied through the lateral channels as sheath flows. The introduction of reagents was precisely controlled using syringe pumps (KF technology, Italy). All chemical solutions were loaded into 5 mL glass syringes (Astech) and delivered to the microfluidic chip inlets via 1/32″ OD × 0.32 mm ID PTFE tubing (Darwin Microfluidics).

To modulate NP properties, the FRR between the central and sheath flows was systematically varied while maintaining a constant TFR. Specifically, three distinct FRRs were investigated. The flow parameters used for AuNP synthesis are summarised in Table 3. The FRR and TFR were defined as follows:

$$\mathbf{F}\mathbf{R}\mathbf{R}=\frac{{\mathbf{Q}}_{\mathbf{c}}}{{\mathbf{Q}}_{\mathbf{s}}}\mathbf{T}\mathbf{F}\mathbf{R}={\mathbf{Q}}_{\mathbf{c}}+{2\mathbf{Q}}_{\mathbf{s}}$$

where Q_c_ is the volumetric flow rate of the central HAuCl₄·3H₂O solution, and Q_s_ is the flow rate of the sheath (HEPES:Na₂HPO₄) solution in each side channel.

**Table 3:** Experimental flow parameters for AuNP synthesis. The table summarises the volumetric flow rate of the central HAuCl₄·3H₂O solution (Qc), the flow rate of the sheath solution comprising HEPES buffer and Na₂HPO₄ in each side channel (Qs), the HEPES:Na₂HPO₄ ratio, the flow rate ratio (Qc:Qs), and the total flow rate

No	HEPES:Na_2_HPO_4_ Ratio	Qc	Qs	Flow Rate Ratio	Total Flow Rate
		(uL/min)	(uL/min)	(FRR)	TFR (uL/min)
1	1:3	30	60	0.5	150
2	1:3	50	50	1	150
3	1:3	75	37.5	2	150

#### Gold nanoparticle characterisation

##### UV–visible spectrophotometry

UV–Visible absorption spectra were collated using a SHIMADZU UV-2600 spectrophotometer. Measurements were conducted over the wavelength range of 400–800 nm. Spectra were recorded at defined time intervals following NP synthesis, specifically at 30 min, 24 h, 72 h, and 7 days post-synthesis, to assess the temporal stability of the AuNPs. All measurements were performed at ambient temperature, using quartz cuvettes with a 1 cm path length. The spectral data were analysed to monitor changes in the surface plasmon resonance band, providing insight into the colloidal stability and potential aggregation of the AuNPs over time. All measurements were performed three times to ensure reliability of the results.

##### Dynamic light scattering

Dynamic light scattering (DLS) measurements were conducted using a Malvern Zetasizer Ultra instrument to determine the hydrodynamic diameter and polydispersity index (PDI) of the AuNPs. Before analysis, 100 μL aliquots of each colloidal AuNP sample were diluted with 900 μL of DI water to minimise multiple scattering effects and ensure optimal measurement conditions. The diluted suspensions were transferred to DTS0012 disposable 10 × 10 mm polystyrene cuvettes for analysis. Each measurement was performed at a fixed acquisition time of 60 s, with three independent replicates acquired (n = 3) per sample to assess reproducibility.

##### Scanning electron microscopy

Surface and morphological characterization of the synthesized AuNPs was performed using a Hitachi S-5500 field emission scanning electron microscope (FE-SEM). Samples were prepared by drop-casting a colloidal suspension of AuNPs, dispersed in a 1:1 (v/v) mixture of ethanol and DI water, onto 300-mesh copper grids (Agar Scientific). The grids were subsequently air-dried at ambient conditions for several minutes to ensure solvent evaporation and particle immobilisation. Imaging was conducted at accelerating voltages ranging from 120 to 200 kV, with an electron beam current between 10 and 15 μA.

#### Catalytic reduction of 4-nitrophenol

**Fig. 3:**
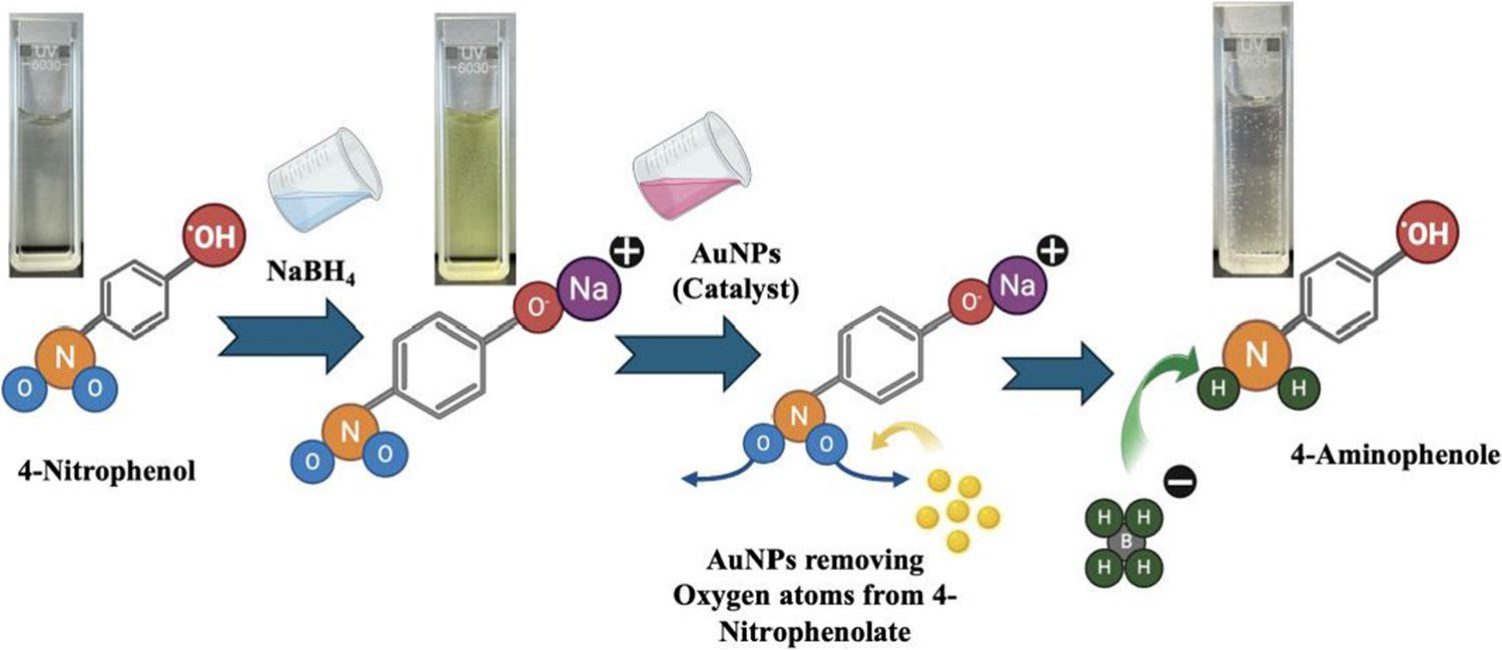
Schematic representation of the catalytic reduction of 4-nitrophenol (4-NP) to 4-aminophenol (4-AP) facilitated by gold nanoparticles (AuNPs). In the presence of sodium borohydride (NaBH₄), 4-NP is first converted to 4-nitrophenolate. AuNPs then catalyze the transfer of hydrogen atoms from the borohydride (BH₄⁻) to the nitro group, reducing it to an amine. The reaction is visually indicated by a distinct color change in the solution, confirming the conversion to 4-AP

The catalytic activity of AuNPs was evaluated via the reduction of 4-nitrophenol (4-NP) to 4-aminophenol (4-AP) [38], a transformation of significant interest due to its relevance in both environmental remediation and fine chemical synthesis. Impact of AuNPs size on the catalytic activity has also been evaluated. Initially, 100 µL of a 100 mM NaBH₄ solution was introduced to 2 mL of a 0.1 mM 4-NP solution, resulting in an immediate color transition from pale yellow to intense yellow, indicative of the formation of 4-nitrophenolate ions under alkaline conditions. UV–vis absorption spectra recorded at this stage exhibited a pronounced peak at 400 nm, characteristic of the 4-nitrophenolate ion. Subsequently, 100 μL of the AuNP suspension with concentration of 0.25 mM was introduced to initiate the catalytic reduction. Concentration of AuNPs in NPs suspension is measured by measuring the original gold solution amount supplied in central inlet compared to the total volume of the suspension. The progress of the reduction was monitored in situ by UV–Vis spectroscopy. All catalytic activity tests are performed 3 times to ensure replication. Figure 3 shows the schematic diagram of this AuNPs induced catalytic activity.

## Results and analysis

### Performance evaluation of multi-material 3D-printed microfluidic connectors

Multimaterial 3D-printed connectors demonstrated excellent performance across all leakage tests conducted under varying flow rates, pressures, and solvent compatibility conditions. The connectors demonstrated no observable leakage and damage when tested with dyed water at pressures up to 5 bar over a continuous 3-h period (Fig. 1S). When tested with air, the connectors maintained integrity up to 1 bar for the same duration. No leakage was observed even under continuous flow operation at extremely high flow rates of 6000 µL/min. The connectors maintained their dimensions over time (Fig. 3S), and no visible breakage or tearing was observed after five reuse cycles. The results are summarized in Table 2S and 3S.

Although most parts of the connectors do not directly contact chemical reagents (with the exception of the lower portion of the male plug’s ferrule), all materials employed in this study exhibited compatibility with a wide range of solvents e.g. Water, Acetonitrile, and Ethanol. No deformation has been noted for both materials till 5 h. After 5 h Tangoblack cube showed slight deformations around the edges for Acetonitrile and Ethanol. VeroClear showed higher overnight chemical compatibility with all the solvents. Both Tangoblack and VeroClear demonstrated perfect compatibility by showing not even a little deformation for 48 h with aqueous solution of reducing agents involved in AuNPs synthesis. A normal NPs synthesis process requires maximum a few minutes in microfluidic chip, (~ 6.67 min in our method to get 1 ml suspension of NPs as TFR is 150ul/min) so these 3D printed connectors have been proven compatible with the solvents used in NPs synthesis. Based on solvent compatibility it can be concluded that these 3D printed connectors can perform perfectly with all type of solvents till 5 h constantly. For prolonged usage (more than 5 h constant usage) these 3D printed can be used with aqueous solvents.

To evaluate the reusability of the 3D printed connectors, all experiments described in Sections "Investigation of connector leakages under different flow conditions" and "Investigation of connector leakages under different pressure conditions" were repeated five times using the same connectors. After each trial, the connectors were carefully unscrewed from the microfluidic chip, washed using Prestige Autoclave classic medical 2100 and examined for any visible signs of wear or mechanical damage. Particular attention was given to the O-rings of the mating ports that serve as gasket and the ferrules of the male adapters, as these components are made of soft tangoblack material and critical for ensuring tight sealing and leak free operation. The inspection involved checking for visible cracks, deformation, or loss of elasticity that might compromise connector performance in subsequent cycles. Once inspected, the connectors were reattached to the chip and subjected to the next round of testing under identical experimental conditions. For the consecutive five repeated cycles of reuse and washing, the connectors consistently maintained their sealing integrity without any major evidence of material fatigue, leakage, or structural failure except slight surface deformation was seen on the gasket of the matting port as can be seen in Fig. 3S.

After the reliability testing under extreme conditions these connectors were integrated into multiple independent AuNPs synthesis runs, encompassing three distinct flow rates mentioned in Table 3, with each condition replicated in triplicate to ensure reproducibility. Throughout all experimental runs of AuNPs synthesis, no leakage, either major or minor, was observed at any interface, indicating robust sealing performance under varying flow conditions. This leak-free operation underscores the connectors’ reliability for continuous-flow NP synthesis applications, where system integrity is critical for reproducibility and contamination avoidance.

Overall, these multimaterial 3D-printed connectors represent a faster, cost-effective, and highly adaptable solution for a wide range of microfluidic applications where reliable connections between platforms and reagent supplies are essential. Their ability to provide precise alignment, robust sealing, and reusability directly addresses the limitations of conventional microfluidic approaches. By enabling tight, detachable, and reproducible sealing, these connectors ensure stable flow conditions and minimize flow disturbances or assembly errors, both of which are critical for maintaining consistency in different microfluidic applications. The use of additive manufacturing further enhances their value, allowing rapid prototyping and customization for diverse chip geometries and materials, thereby offering flexibility for experimental design and seamless adaptation to different synthesis conditions. To demonstrate their practical performance, NPs synthesis was chosen as a representative application, given its strong dependence on flow uniformity and reproducibility; the results highlight how these connectors support stable operation, consistent outcomes, and improved reproducibility. Moreover, their low-cost, on-demand production reduces reliance on expensive commercial alternatives, making them a scalable and accessible solution that advances the efficiency, adaptability, and reliability of microfluidic systems.

### Microfluidic-based synthesis of AuNPs

All reactions for AuNPs synthesis were conducted with glass reservoir syringes loaded on syringe pumps (KF technology, Italy) attached to the PMMA and PSA based microfluidic chip. AuNPs formation was observed with the naked eye within the outlet tubing and in the collection cuvette, with complete synthesis achieved in approximately less than 2 min. In a previous study conducted by the author Bilal et al. [35] the conventional batch synthesis of AuNPs was performed using HAuCl₄ and HEPES buffer in the presence of Na₂HPO₄. Typically, this disproportionation (redox) reaction required approximately 10 to 15 min and was carried out in disposable plastic cuvettes at room temperature. Bilal et al. batch synthesis method involved seedless chemical synthesis of AuNPs using a combination of HEPES and Na₂HPO₄ under varying physicochemical conditions, including temperature, pH, and reactant concentrations. In this synthesis, HEPES acts as the primary reducing agent (Au^3^⁺ → Au⁰) and capping/structure-directing ligand. HEPES-derived free radicals adsorb onto nascent Au nuclei surfaces, guiding the anisotropic growth of the AuNPs and preventing the aggregation of gold nuclei. Na₂HPO₄ provides the highly reactive phosphate ions that conjugate with the HEPES-derived free radicals on the AuNPs surface, clustering into polymeric corona chains that further passivate the surface and suppress secondary growth. Consequently, the HEPES and Na₂HPO₄ molar ratio, along with pH and temperature, controls the nucleation and growth kinetics, particle size/shape, and the evolution of the LSPR band.

**Fig. 4:**
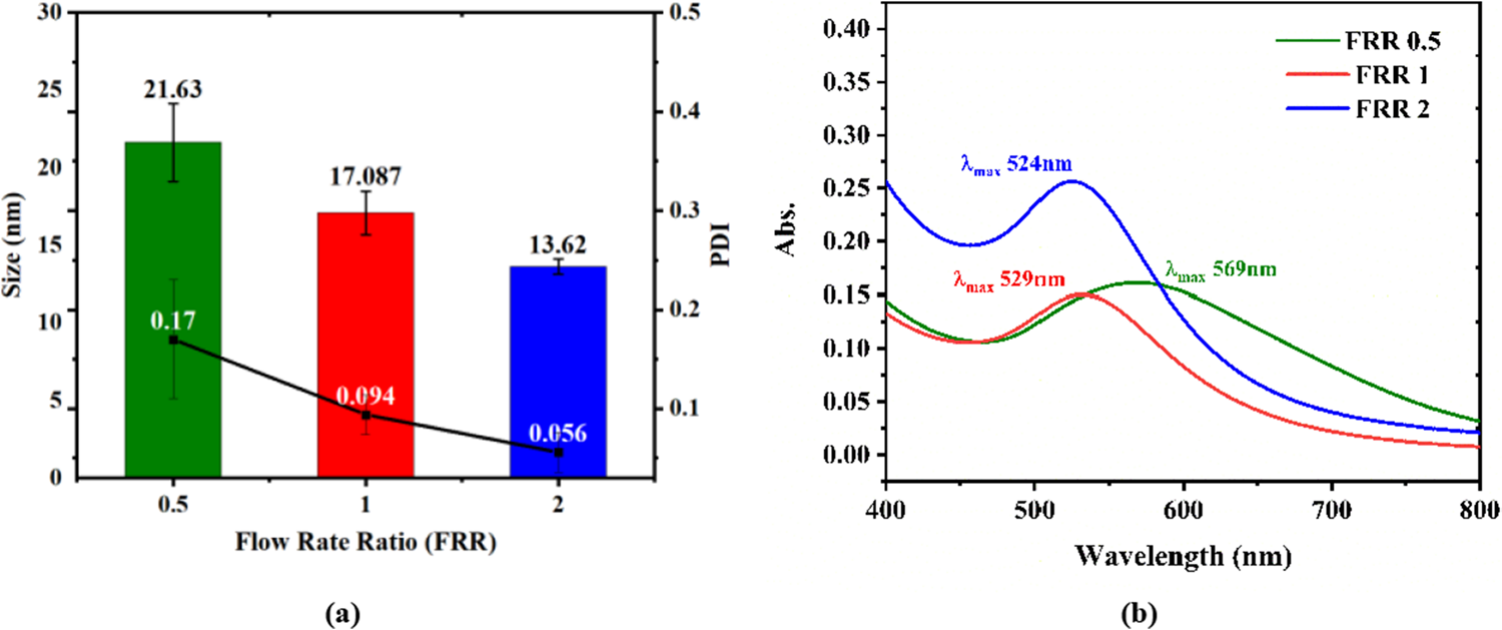
Characterization of AuNPs synthesized under different FRR a) Average particle size and polydispersity index (PDI) of AuNPs as a function of FRR, measured by dynamic light scattering (DLS), b) UV Vis absorption spectra of AuNPs synthesized at FRR

This approach led to AuNPs with diverse morphologies and a broad size distribution ranging from less than 1 nm up to around 100 nm. The process depended significantly on temperature and pH, influencing particle nucleation, growth rates, and morphologies, including spherical, anisotropic, and flower-like structures. AuNPs displayed SPR peaks varying between 528 and 593 nm, indicating substantial variations in particle size and shape distributions [35, 36]. Such bulk methods, however, often lead to inconsistent mixing efficiencies and heterogeneous nucleation, resulting in broad particle size distributions, lower reproducibility, and reduced control over NPs. However, the microfluidic assisted synthesis of AuNPs in this study represents a significant reduction in reaction time relative to conventional batch synthesis methods, which typically require substantially longer durations for NP formation. It enables precise control over AuNPs by only regulating FRR. Overall microfluidic approach presented in this study produced AuNPs with narrower, highly uniform size distributions, as confirmed by UV–Visible spectroscopy and DLS under all three FRR (0.5, 1 and 2) as mentioned in Fig. 4.

These results illustrate superior reproducibility and consistency compared to the batch synthesis approach. The microfluidic method significantly reduces reaction times to seconds or minutes, leveraging rapid, uniform mixing in microchannels, whereas the batch method required longer incubation periods (up to 15 min) and had lower reproducibility due to less consistent mixing dynamics. Additionally, the microfluidic setup facilitates scalability through reducing reagent usage and waste, offering a clear advantage over traditional batch methods in terms of economic and environmental sustainability. Hence, microfluidics offers a tunable and controllable approach for AuNPs synthesis as it provides substantial improvements in precision, reproducibility, scalability, and particle uniformity; thus presenting enhanced practicality and suitability for advanced biomedical and industrial applications.

#### Synergistic influence of flow rate ratio on gold nanoparticle properties

**Fig. 5:**
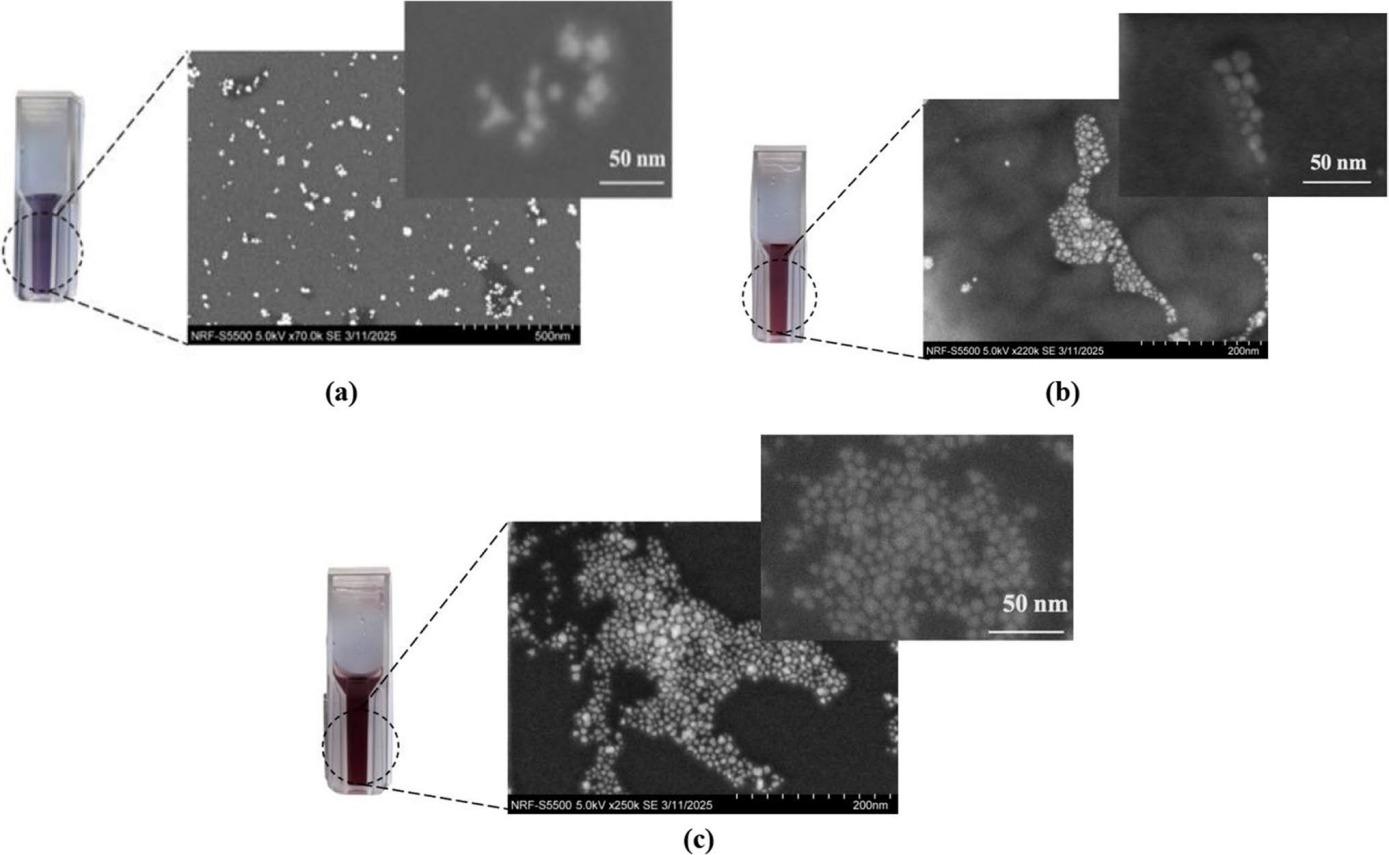
Morphology of AuNPs analyzed through an SEM microscope. Different FRR leads to different colors of AuNPs colloidal suspensions. At the lower FRR, AuNPs secondary growth is evident, while a shift from star-shaped AuNPs to more spherical AuNPs can be observed with an increase in FRR. a) FRR 0.5 (21.63 ± 2.5 nm), b) FRR 1 (17.09 ± 1.4 nm), c) FRR2 (13.62 ± 0.5)

The present study systematically investigates the effect of varying FRR within a microfluidic platform on the optical and physical characteristics of AuNPs. By modulating the FRR, AuNPs with diameters ranging from 21.63 ± 3 to 13.62 ± 0.5 nm are synthesised as can be seen in Fig. 4a. It demonstrate a pronounced dependence of AuNPs size, PDI (Fig. 4a), and UV–Vis absorption spectra on FRR (Fig. 4b). These findings depicts that adjustment of the FRR enables precise control over AuNP nucleation and growth kinetics, as reflected in the observed shifts in the size and PDI values (Fig. 4a) as well as UV–Vis absorption maxima (Fig. 4b), without necessitating the alteration of other synthesis variables such as pH, reactant concentrations, or the stoichiometry of reducing and capping agents while synthesizing AuNPs using batch approach [35, 36, 39]. Thus microfluidic approach offers enhanced reproducibility and tunability relative to conventional batch methods. Figure 4 depicts a very clear trends in size distribution, PDI, and maximum absorbance peak position as a function of FRR [40]. Morphological analysis further demonstrates the differences in NP shape with FRR, that highlights its critical role in controlling the AuNPs synthesis mechanism (Fig. 5). Table 4 depicts a concise summary of AuNPs physiochemical properties (size, PDI, λ_max, and morphology) assessed under different FRR ranging from 0.5 to 2.

**Table 4:** Size, polydispersity index (PDI), and λ_max_ of AuNPs synthesized at different flow rate ratios (FRR)

FRR	Size (nm)	PDI	Zeta Potential	λ_max_ (nm)
0.5	21.63 ± 2.5	0.17 ± 0.06	−21.01 ± 3.32	569
1	17.09 ± 1.4	0.095 ± 0.02	−22.4 ± 2.4	529
2	13.62 ± 0.5	0.056 ± 0.01	−24.74 ± 4.42	524

Varying the FRR in microfluidic synthesis of AuNPs exerts dual and opposing effects on the reaction environment, significantly influencing particle size and morphology. An increase in FRR while maintaining a constant TFR reduces the relative amount of the HEPES stream, thereby decreasing overall HEPES concentration in the reaction zone. At the same time, a higher FRR leads to reduced mixing efficiency between the precursor streams, which is also a critical factor in NPs formation. Decrease in FRR enhances mixing efficiency. Higher sheath flow rates as compared to the central stream flow rates result in narrower and focused central stream, which reduces the diffusion distance between fluids and accelerates mixing [41, 42].

## FRR impact on mixing behavior in microchannel

The mixing behavior within our rectangular microchannel, governed by hydrodynamic flow focusing, is an interplay between molecular diffusion across the interfaces of co flowing streams and advective transport along the channel length due to high flow velocities. To quantitatively assess this process, we first characterized the flow regime. The Reynolds number (Re), which represents the ratio of inertial to viscous forces, was calculated under the applied flow conditions as:

$$Re=\frac{{\rho vD}_{h}}{\mu }$$

where *ρ* is the fluid density (1000 kg/m^3^ for water), v is the characteristic velocity at the center of the channel (0.0485 m/s), *μ* is the dynamic viscosity (1 mPa.s for water), D_h_ is the hydraulic diameter of the rectangular channel can be calculated as ${(D}_{h}=\frac{2wd}{w+d}$). Our calculations confirmed that Re remained 8.22 for all three flow conditions, firmly establishing laminar flow with the absence of turbulent mixing.

The Peclet number (Pe), which defines the ratio of advective to diffusive transport rates:

$$\mathrm{Pe}=\frac{{\mathrm{vL}}_{\mathrm{c}}}{\mathrm{D}}$$

Here, L_c_ corresponds to the width of the focused stream (w_f_), which increases with the flow rate ratio (FRR). It is calculated as: (w_f_ = w $\frac{{\mathrm{Q}}_{\mathrm{c}}}{\mathrm{TFR}})$, where w is the channel width [43]. The diffusion coefficient of the aqueous solutions was taken as D ≈2.3 × 10^–9^ m^2^/s. The calculated Peclet numbers were 2530 (FRR = 0.5), 4217 (FRR = 1), and 6326 (FRR = 2), all much greater than unity. This confirms that mixing does not occur primarily at the junction of the co-flowing streams but rather develops along the channel length in direction of flow.

Based on this it can be concluded that the flow regime in our rectangular microchannel can be conclusively described as laminar (low Re) with mixing happened along the direction of flow, highlighting that hydrodynamic flow focusing promotes interface formation while efficient transport is primarily governed by advection due to high Peclet numbers. The 2- Dimension flow focusing model of mixing time defines that mixing time increases with increase in FRR [44]. Hence with lower FRR we can get better mixing conditions in our microchannel.

## FRR impact on intrinsic kinetics of the reduction reaction

On the other hand, HEPES functions both as a mild reducing agent and a capping ligand; therefore, its concentration plays a key role in the nucleation and growth dynamics of AuNPs [45]. Recent studies have shown that increasing HEPES concentration leads to the formation of larger particles because of the formation of longer and thicker branches on AuNPs, leading to more anisotropic (non-spherical) growth. The increase HEPES increases the density of branches attached to the cores [40, 46]. This morphological shift is typically accompanied by a red shift in the SPR peak, reflecting increased size [40, 46–51]. Increase the buffer (HEPES) to precursor (HAuCl₄) concentration ratio that happened when we decrease the central stream flow rate as compared to side stream flow rate i.e. decrease in FRR, significantly impact on the growth mechanism of AuNPs. Higher FRR with lower HEPES concentration promotes controlled, seed-mediated-like growth, resulting in AuNPs with well-defined cores and shorter, more uniform branches [40]. These particles are more monodisperse and display localized surface plasmon (LSP) bands at shorter wavelengths [40]. On the other hand, at lower FRR with same TFR, HEPES concentration increases. The excess of HEPES enhances both the reduction rate of gold ions and the selective capping of certain crystal planes. This leads to the formation of AuNPs with longer and denser protrusions, causing a red shift in the SPR band due to increased branch length and stronger plasmon between the center of the particle and its branches [46]. At high HEPES concentrations, the growth becomes too rapid to allow distinct seed formation, favoring seedless like mechanisms that yield heterogeneous NPs with small or poorly defined cores and irregular branch lengths. This shift in growth dynamics results in further red shifting of the SPR and increased polydispersity [40, 46].

Taken together, these results suggest that the effect of FRR on AuNP formation arises from the interplay between mixing kinetics and reduction kinetics. At lower FRR, enhanced mixing efficiency reduces diffusion distances, enabling more rapid homogenization of reactants and promoting diffusion limited nucleation. In contrast, at higher FRR the narrower focused core reduces HEPES concentration in the reaction zone, which leads towards smaller sized NPs. The final AuNPs size, shape, and dispersity results from a balance between these two opposing influences i.e. diffusion limited mixing versus reaction driven growth. This highlights FRR as a critical parameter for tuning AuNPs synthesis in microfluidic systems. Table 4 provide a concise summary of AuNPs properties synthesized under different FRR.

## Effect of flow rate ratio on AuNP size and polydispersity index

The average hydrodynamic particle size was determined from the z-average obtained via Dynamic Light Scattering (DLS) analysis (Fig. 4a). Figure 4 shows the mean values with standard deviations calculated from the three independent trials for each experimental condition. The influence of FRR on the hydrodynamic diameter and PDI of AuNPs was systematically investigated by varying the FRR from 0.5 to 2. Modulation of the FRR resulted in pronounced changes in both particle size and PDI. Specifically, increasing the FRR led to a reduction in average particle diameter from 21.62 ± 3 to 13.62 ± 0.5 nm, accompanied by a decrease in PDI from 0.168 ± 0.065 to 0.056 ± 0.0064, indicative of enhanced size uniformity which can be clearly seen in Fig. 4a.

The FRR within the microchannels governs two critical parameters: the efficiency of reagent mixing and the temporal delivery of reactants to the reaction zone. These factors collectively modulate the nucleation rate and the reduction kinetics of Au3⁺ to Au°, which are responsible for the resulting NP size distribution.

Elevated FRR values facilitate in accelerating nucleation and promoting the formation of a greater number of nuclei within a shorter residence time. This rapid nucleation restricts subsequent particle growth, yielding smaller and more monodisperse NPs. Conversely, lower FRR values are associated with prolonged growth phases, resulting in larger particles with broader size distributions.

DLS was used as the primary technique for determining the hydrodynamic diameter and PDI of the synthesised AuNPs. To verify the DLS data and assess particle morphology, SEM was performed. The convergence between DLS and SEM analysis confirms the reliability and reproducibility of the microfluidic synthesis approach for generating AuNPs with tuneable and well-defined physicochemical properties.

In our microfluidic setup AuNPs are synthesized via HFF. Initially, the central microchannel introduces an aqueous solution of chloroauric acid (HAuCl₄·3H₂O), which is a source of Au(III) ions. Simultaneously, premixed sheath solutions containing HEPES buffer and Na₂HPO₄ flow from the lateral inlets at a precise 45° angle, creating a laminar sheath around the central gold precursor stream. This synthesis mechanism initiates at the junction region, the intersection of central and lateral channels, where controlled laminar mixing rapidly brings reagents into intimate molecular contact. At this interface, HEPES acts as the primary reducing agent, donating electrons to the Au(III) ions, progressively reducing them first to Au(II) intermediates and subsequently to Au(I). The Au(I) species are further reduced to elemental gold (Au°) NPs, forming nucleation sites. Meanwhile, this reaction generates cationic HEPES free radicals, which adsorb onto and stabilize the freshly formed gold nuclei, preventing particle aggregation and controlling particle growth.

Additionally, the presence of Na₂HPO₄ plays a crucial chemical role: phosphate ions generated from disodium phosphate interact strongly via hydrogen bonding with the terminal oxygen groups of the HEPES radicals, forming a stabilizing polymeric corona on the nanoparticle surface. This polymeric corona effectively halts uncontrolled secondary growth, thus providing enhanced particle stability and narrower size distributions.

As the fluid stream progresses downstream through the 600 µm-wide main channel, the reaction continues under highly reproducible hydrodynamic conditions. At higher flow rate ratios (FRR 2), the reaction conditions leads to very small. Conversely, at lower values of FRR (0.5 & 1), the reaction conditions yields the larger and broader size distributions as indicated by broader DLS peaks and lower UV-absorption intensities. As previously discussed, under constant TFR of 150ul/min, increasing FRR from 0.5 to 2 results in a relative decrease in HEPES concentration, which in turn can favor the formation of smaller AuNPs from 21.62 ± 3 to 13.62 ± 0.5 nm due to the kinetic and surface effects associated with lower HEPES levels. On the other hand, decreasing the FRR enhances mixing efficiency within the microchannel, which promotes rapid nucleation and generally yields smaller, more monodisperse nanoparticles. Notably, at FRR of 0.5, despite the relatively high concentration of HEPES, these enhanced mixing conditions dominate, resulting in only a modest increase in AuNP size (1.62 ± 3).This suggests that mixing efficiency can partially offset the particle enlarging effects of elevated HEPES concentration[40–42, 48, 49].

## Effect of FRR on the surface plasmon resonance of AuNPs

The FRR during NP synthesis was found to influence the localised surface plasmon resonance (LSPR) characteristics of the resulting AuNPs. UV–Vis absorption spectroscopy was employed to characterise the optical properties of the AuNP dispersions. With the increase in FRR from 0.5 to 2 the absorption maxima (λ_max_) of the AuNPs exhibited a systematic blueshift with decrease in particle diameter, consistent with established plasmonic behaviour.

Specifically, AuNPs with an average diameter of 21.62 ± 3 nm displayed an LSPR peak at 569 nm, whereas smaller NPs with mean diameters of 17.08 ± 1.45 nm and 13.62 ± 0.5 nm exhibited LSPR peaks at 529 nm and 524 nm, respectively as shown in Fig. 4b, This trend aligns with the theoretical understanding that larger AuNPs support plasmon oscillations at longer wavelengths due to increased electron oscillation path lengths, resulting in a redshift of the LSPR band [52] as well as due to the amount of reductant vs precursor [45]. Among the tested flow rates, FRR 2 (blue line) exhibits the highest absorption intensity, indicating enhanced nanoparticle synthesis efficiency as compared to FRR 1 and 0.5. FRR 1 (red line in Fig. 4b) demonstrates the lower peak intensity or the absorbance of the UV–Visible light, suggesting fewer nanoparticles. FRR 0.5 (green line in Fig. 4b) shows lowest absorbance with a very broad peak area, representing large size of nanoparticle formation or potential aggregation, making comparatively less efficient condition. It is because that varying FRR from 0.5 to 2 leads towards decrease in HEPES concentration that in turn leads to the blue shift of the main LSPR peak of the AuNPs [40, 47–49]. This blueshift also depicts that adjusting the flow rate ratio significantly impacts the efficiency of AuNPs synthesis. The highest absorption observed at FRR 2 indicates optimal synthesis conditions, beneficial for producing stable and well-defined AuNPs through microfluidic techniques. This corelates very well with the DLS result for FRR 2, showing uniform and small sized AuNP, which typically exhibit sharp and intense SPR absorption peaks. FRR 1 displayed lower absorbance in UV–visible spectra, corresponding with the intermediate particle size and narrower distribution observed by DLS. The smaller size and relatively uniform distribution contribute to the lower absorption intensity observed. FRR 0.5 presented moderate UV–visible absorbance, reflecting larger nanoparticles with a broader size distribution, typically yielding slightly less defined SPR peaks through UV–Visible spectra due to increased polydispersity and particle aggregation tendencies.

Repeated UV–Vis measurements over time demonstrated no significant changes in the absorption spectra, indicating that the synthesised AuNPs remained colloidally stable under the storage and measurement conditions employed. The persistence of a sharp and well-defined LSPR peak further supports the stable and monodispersed AuNPs suspensions.

## Effect of FRR on the morphology of AuNPs

The morphological impact of FRR is clearly visible in Fig. 5. At higher FRR values, where the flow rate of the gold precursor (HAuCl₄) is higher relative to the buffer solution (HEPES and Na₂HPO₄), the resulting AuNPs are predominantly spherical as can be seen in Fig. 5c. This occurs because a higher precursor concentration promotes faster, isotropic growth, and the relatively lower concentration of buffer molecules provides less shape control, leading to compact, round particles.

In contrast, by decreasing FRR (i.e., as the buffer concentration increases relative to HAuCl₄), the morphology shifts toward branched nanoparticles [40, 46]. This trend can be clearly seen in Fig. 5a which shows SEM image of AuNPs synthesized at FRR 0.5 growth of small protrusions can be easily seen while as we move towards FRR 1 and 2, an evident shift towards spherical shape AuNPs can be clearly seen in Fig. 5b and c. This happens when fewer HEPES molecules are available to bind to the surface of growing gold nanoparticles, leaving various crystal surfaces exposed for further Au3⁺ reduction. As the reaction proceeds, more Au3⁺ ions are reduced on the nanoparticle surface, resulting in the formation of spherical particles with short protrusions due to relatively uniform growth across multiple planes. At lower FRR with the excess of HEPES buffer available, its molecules bind preferentially to certain crystallographic planes, while exhibiting weak or negligible adsorption on the {111} planes—a family of densely packed, low-energy surfaces characteristic of face-centered cubic (FCC) structures. These planes are thermodynamically stable and less reactive, which allows them to remain exposed during synthesis. As a result, continued Au3⁺ reduction promotes directional growth along the ⟨111⟩ axis, leading to the formation of multi-branched gold nanostructures. Furthermore, since HEPES also acts as a mild reducing agent, higher HEPES concentrations accelerate Au3⁺ reduction specifically on the exposed {111} planes, resulting in longer and more pronounced protrusions [46].

### Catalytic activity of synthesised AuNPs

**Fig. 6:**
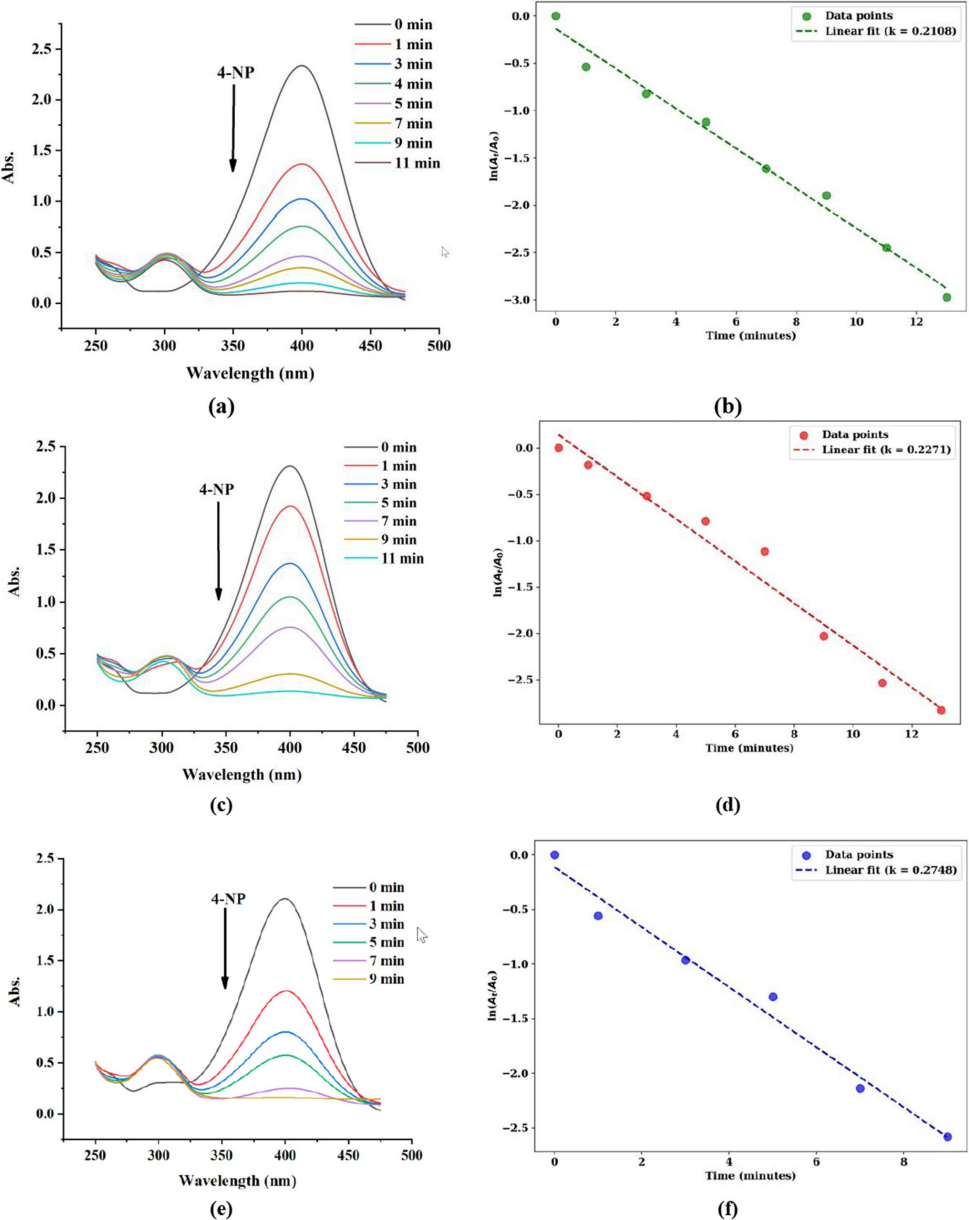
UV–Vis absorption spectra and corresponding pseudo-first-order kinetic plots for the catalytic reduction of 4-nitrophenol (4-NP) using AuNPs synthesized at different flow rate ratios (FRR): (**a**) spectra for AuNPs synthesized at FRR 0.5, (**b**) kinetics for AuNPs synthesized at FRR 0.5, (**c**) spectra for AuNPs synthesized at FRR 1, (**d**) kinetics for AuNPs synthesized at FRR 1, (**e**) spectra for AuNPs synthesized at FRR 2, and (**f**) kinetics for AuNPs synthesized at FRR 2

These AuNPs synthesized on the microfluidic platform are employed for catalytic reduction of 4-NP to the 4-AP. In the absence of AuNPs, the absorbance at 400 nm remained essentially unchanged over time, confirming the lack of spontaneous reduction of 4-NP by NaBH₄ alone. Subsequent addition of 100 µL of AuNP suspension initiated catalytic reduction of 4-NP. The 4-NP ions adsorbed onto the AuNP surfaces, where they underwent reduction to 4-aminophenol (4-AP) via electron transfer from hydrogen species generated by NaBH₄ [38]. This transformation involves the substitution of the nitro group’s oxygen atoms with hydrogen atoms, yielding 4-AP as the product. The progress of the reaction was monitored by the gradual decrease in absorbance at 400 nm, with the emergence of a new absorption band near 300 nm, corresponding to 4-AP formation as can be seen in Fig. 6a,c and d.

It is evident from Fig. 6a that the gradual decrease in the absorption peak at ~ 400 nm, corresponding to the concentration of 4-NP, is relatively slow for AuNPs synthesized at FRR 0.5. This decrease becomes faster for AuNPs synthesized at FRR 1 (Fig. 6c) and further accelerates for those synthesized at FRR 2 (Fig. 6e). The slopes of the linear fits in the ln(At/A0) versus time plots confirm this trend, where A_t_ is the absorbance at time t and A_0_ is the absorbance at t = 0. As shown in Fig. 6b, the rate constant for AuNPs synthesized at FRR 0.5 increases progressively for those synthesized at FRR 1 (Fig. 6d) and FRR 2 (Fig. 6f).

The results are summarized in Table 5, showing that the rate constants increase from 0.2108 min⁻1 (FRR 0.5) to 0.2271 min⁻1 (FRR 1.0), and further to 0.2748 min⁻1 (FRR 2.0). Correspondingly, the half-life decreases from 3.29 min (FRR 0.5) to 3.05 min (FRR 1.0) and 2.52 min (FRR 2.0), indicating that higher FRRs enhance the catalytic conversion of 4-NP. The high correlation coefficients (R2) confirm the excellent linear fits, validating the pseudo first order kinetic model.

**Table 5:** Kinetic parameters of 4-nitrophenol reduction using AuNPs synthesized at different FRRs

AuNPs at FRR	Time for complete reaction (min)	Rate Constant (k) min^−1^	Half-life (t_1/2_) (min)	R^2^
0.5	11	0.210828	3.2877	0.9870
1	11	0.227115	3.0520	0.9719
2	9	0.274826	2.5221	0.9811

The catalytic efficiency AuNPs synthesized at different FRR was systematically investigated. Notably, AuNPs synthesized at FRR 2 exhibited enhanced catalytic activity relative to their larger counterparts as can be seen in Fig. 6. This improvement is attributed to the increased surface area-to-volume ratio of relatively smaller and spherical shaped AuNPs, which affords a greater density of accessible active sites for catalysis. Smaller AuNPs also facilitate more efficient electron transfer between NaBH₄ and 4-NP, thereby accelerating the reduction kinetics. Additionally, reduced diffusion barriers associated with smaller NPs promote improved mass transport, optimizing the interaction between reactants and catalytic surfaces.

In this paper, we demonstrated the catalytic reduction of 4-NP with AuNPs as a proof of concept. However, our platform can be extended to a wide range of microfluidic applications. The system is capable of synthesizing different types of nanoparticles under diverse experimental conditions, offering broad applicability. AuNPs generated on chip could be further surface functionalized with recognition elements such as antibodies or DNA strands for biosensing and diagnostic assays, enabling rapid detection of disease biomarkers. Likewise, environmentally relevant targets such as heavy metals, pesticides, or organic pollutants could be monitored through the plasmonic or catalytic responses of the synthesized nanoparticles.

## Conclusions

This microfluidic architecture addresses key limitations of conventional single-use, adhesive-based connectors by enhancing reusability, cost-efficiency, and sealing performance under elevated pressures. The assemblies exhibited no detectable leakage at liquid pressures up to 5 bar and air pressures up to 1 bar, confirming their suitability for demanding microfluidic applications. Additionally, the connectors maintained consistent performance over at least five reuse cycles, demonstrating a substantial extension in operational lifespan compared to existing solutions.

The 3D-printed plugs and ports also enabled effective investigation of the impact of flow rate ratio (FRR) on gold nanoparticle (AuNP) synthesis. FRR was shown to significantly influence the physicochemical properties of the resulting AuNPs, which directly affect their application-specific performance. For example, smaller AuNPs synthesized at an FRR of 2 displayed greater catalytic efficiency in the reduction of 4-nitrophenol to 4-aminophenol than the larger particles produced at an FRR of 0.5. These findings emphasize the importance of precise fluidic control in tailoring nanoparticle properties for targeted applications.

Overall, the presented 3D-printed connector system provides a robust, reusable, and scalable solution to integration challenges in microfluidic nanoparticle synthesis, offering a promising platform for broader adoption in analytical and nanomaterial workflows.

## Supplementary Information

Below is the link to the electronic supplementary material.

Supplementary file1 (DOCX 3245 KB)

## Data Availability

No datasets were generated or analysed during the current study.
