# Supplementary material for: Tunable gold nanoparticle synthesis using microfluidic flow focusing enabled by reusable 3D-printed multimaterial connectors
Source: Mikrochim Acta. 2026 Feb 19;193(3):169. doi: 10.1007/s00604-026-07848-4 (PMC12920715; doi:10.1007/s00604-026-07848-4)
Supplement: Supplementary file 1 — Supplementary file1 (DOCX 3245 KB) [file 604_2026_7848_MOESM1_ESM.docx]

Supporting Information

Development of Multi-Material 3D-Printed Microfluidic Connectors for Nanoparticle Synthesis Application

Muhammad Mubashar Saeed ^1-4^, Bilal Javed ^5-6^, Eadaoin Carthy ^2-4^, Nicholas Dunne ^2,3,7-13^, David Kinahan ^2,-4^

^1^ML-Labs Centre for Research Training, Dublin City University, Ireland

^2^School of Mechanical and Manufacturing Engineering, Dublin City University, Ireland

^3^Biodesign Europe, Dublin City University, Ireland

^4^RAPID institute, Dublin City University, Ireland

^5^School of Food Science and Environment Health, Technological University Dublin Ireland

^6^Nano Lab Research Centre, Physical to Lifescience Research Hub, Technological University Dublin Ireland

^7^Centre for Medical Engineering Research, School of Mechanical and Manufacturing Engineering, Dublin City University, Dublin, Ireland

^8^Advanced Manufacturing Research Centre (I-Form), School of Mechanical and Manufacturing Engineering, Dublin City University, Dublin, Ireland

^9^SFI Research Centre for Medical Devices (CÚRAM), University of Galway, Galway, Ireland

^10^Advanced Materials and Bioengineering Research Centre (AMBER), Trinity College Dublin, Dublin, Ireland.

^11^School of Pharmacy, McClay Research Centre, Medical Biology Centre, Queen’s University Belfast, Belfast, UK

^12^Department of Mechanical and Manufacturing Engineering, School of Engineering, Trinity College Dublin, Dublin, Ireland

^13^Trinity Centre for Biomedical Engineering, Trinity Biomedical Sciences Institute, Trinity College Dublin, Dublin, Ireland

| 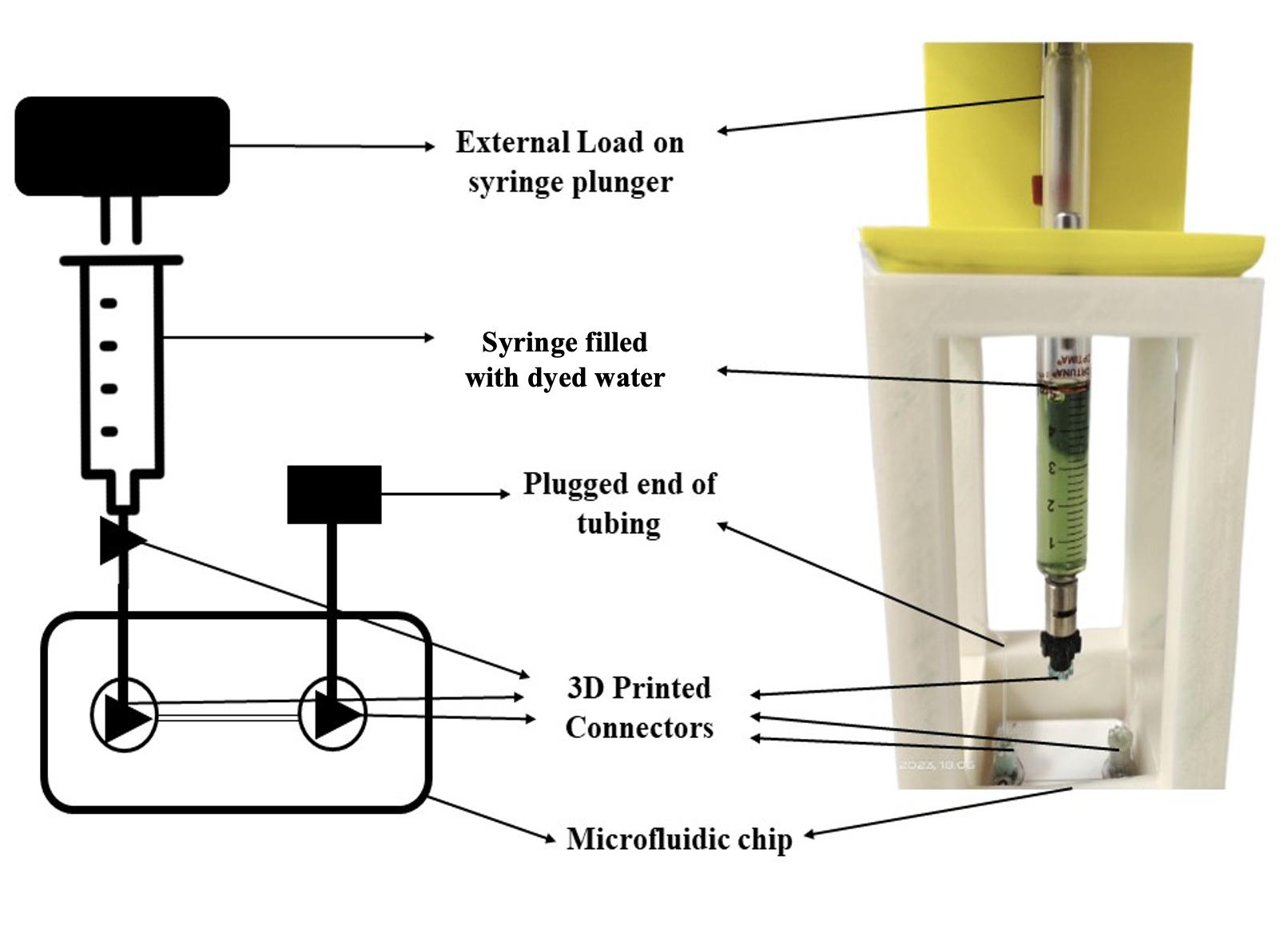 |
| --- |
| Figure 1S*: Investigating the leakage of 3D printed connectors through pressure testing using a custom-designed 3D printed test rig* |

| 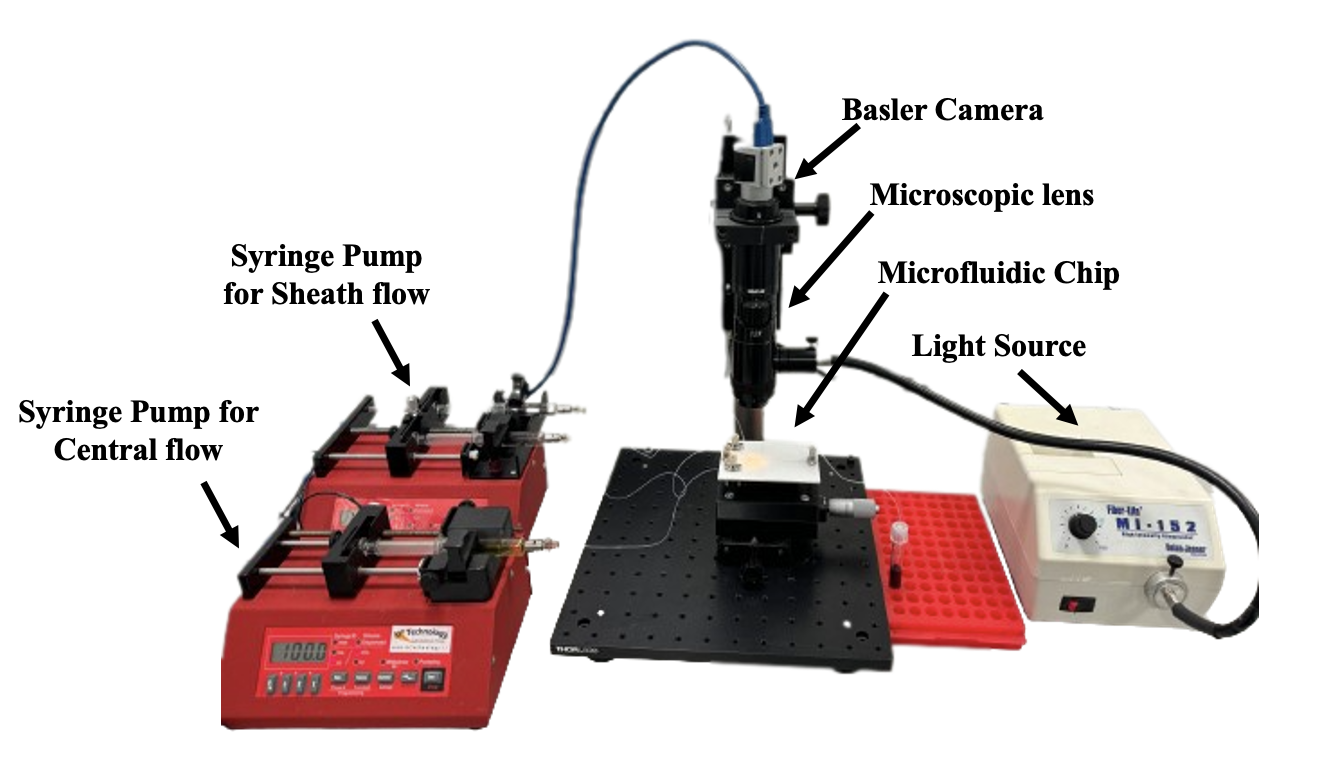 |
| --- |
| Figure 2S: Experimental setup for real-time monitoring of microfluidic nanoparticle synthesis. A Basler camera equipped with a microscopic lens was positioned above the microfluidic chip and connected via Pylon Viewer software to continuously monitor channel conditions and minimize non-uniform flow. This system enabled immediate detection of bubbles or flow disturbances, which were corrected by temporarily applying higher flow rates to flush the channel. To further maintain stable operation and reproducible synthesis conditions, the chip was rinsed with Milli-Q water at a total flow rate of 500 µL/min after every three runs, effectively removing deposited particles and preventing clogging or unstable flow in subsequent experiments*.* |

#

| 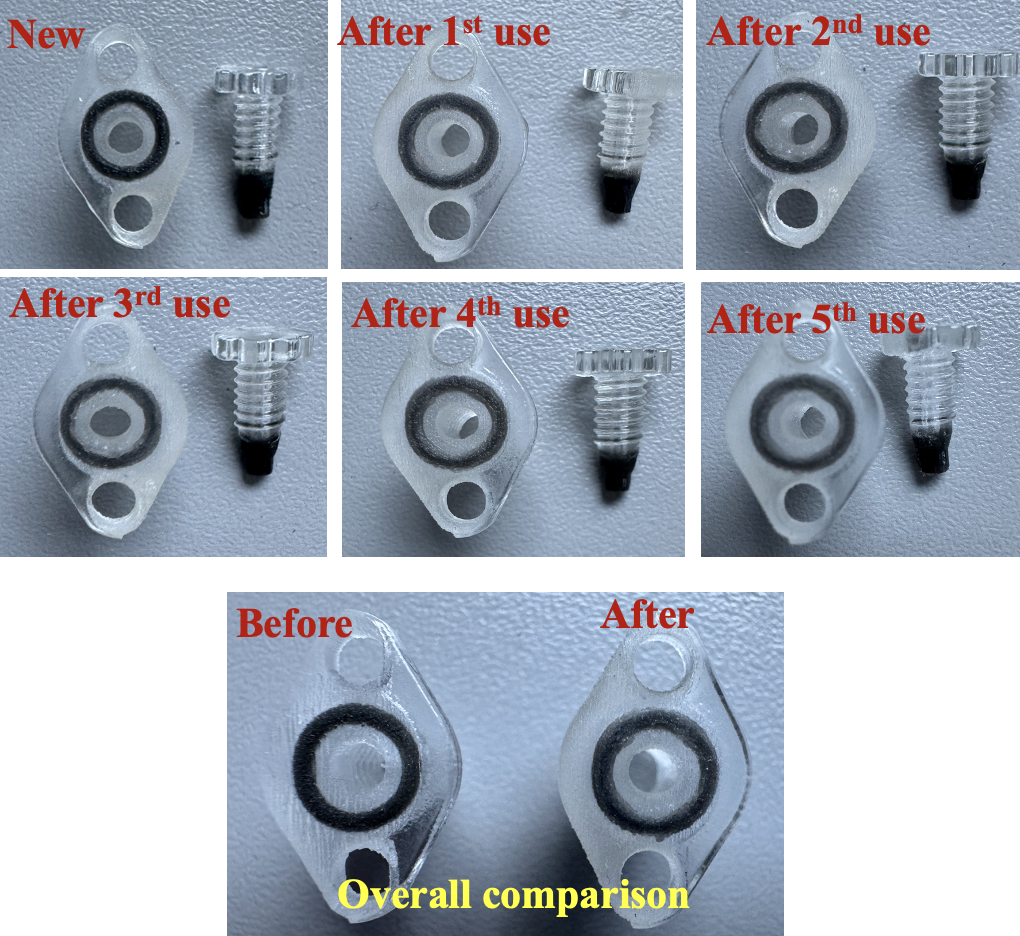 |
| --- |
| Figure 3S Reusability testing of 3D-printed connectors through repeated attachment and detachment cycles. The connector and screw assembly were subjected to five consecutive uses, with visual inspection performed after each cycle to assess sealing integrity and structural durability. Images show the connector in its new condition and progressively after the 1st, 2nd, 3rd, 4th, and 5th uses. Despite repeated tightening and loosening, the sealing ring maintained its shape and functionality, and no visible cracks or deformation were observed in the connector body. The overall comparison (bottom panel) between the connector before use and after five cycles highlights the robustness and reusability of the design, confirming its suitability for multiple experimental runs without compromising performance |

| 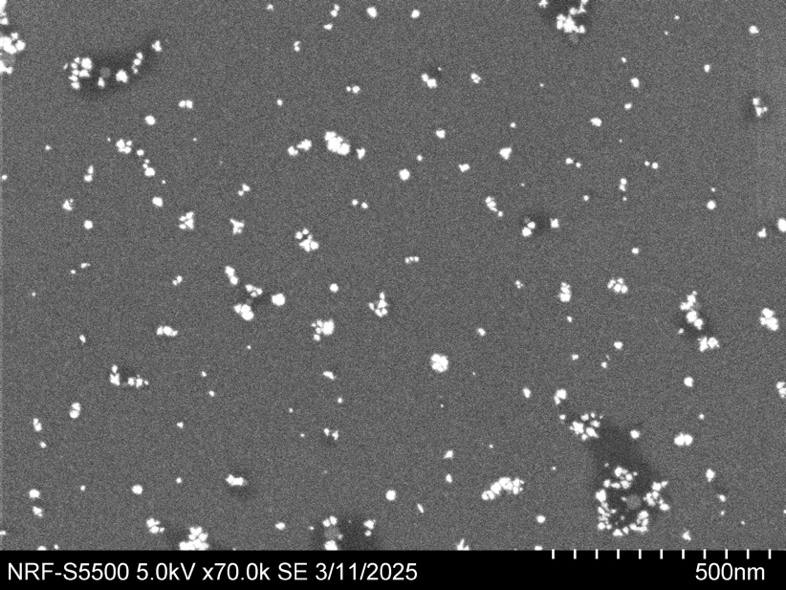 |
| --- |
| Figure 3S*: SEM image of AuNPs synthesized at FRR 0.5* |

| 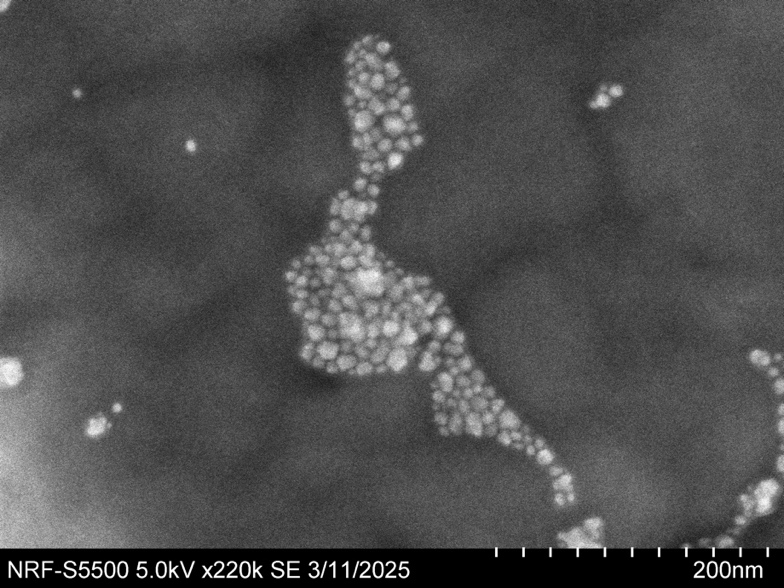 |
| --- |
| Figure 4S*: SEM images of AuNPs synthesized at FRR 1* |

| 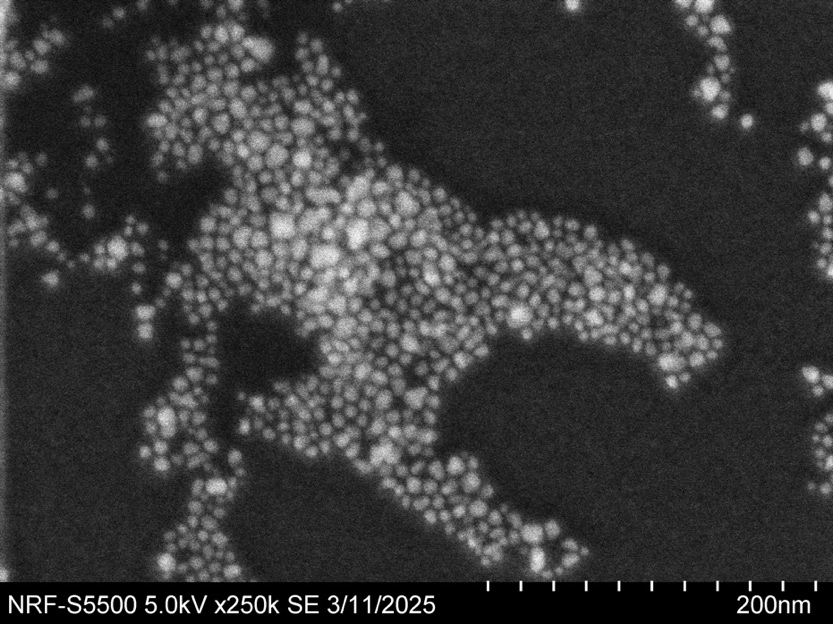 |
| --- |
| Figure 5S*: SEM image of AuNPs synthesized at FRR 2* |

# Table 1S :Comparison of key dimensional measurements between CAD models and fabricated 3D-printed components.

| ***Dimension***  ***(mm)*** | ***Measurement (CAD)*** | ***Measurement***  ***(3D-Print)*** |
| --- | --- | --- |
| *Major Diameter* | *4.3* | *4.3* |
| *Minor Diameter* | *3.5* | *3.5* |
| *Pitch* | *1* | ~*1* |

# Table 2S: Summary of leakage testing parameters for 3D-printed microfluidic connectors. Experimental conditions, including total flow rate, applied pressure, and exposure duration, are reported for each leakage assessment study. Pressure at these total flow rates calculated using Hagen–Poiseuille equation as follows Where Q is the flow rate (in cubic meters per second, m³/s), L is the channel length (in meters, m), w is the channel width (in meters, m), h is the channel depth (in meters, m), and μ indicates the dynamic viscosity (in pascal-seconds, Pa·s).

$$\frac{\boldsymbol{12}\boldsymbol{\mu LQ}}{\boldsymbol{w}\boldsymbol{h}^{\boldsymbol{3}}\left( \boldsymbol{1}-\boldsymbol{0}.\boldsymbol{63}\frac{\boldsymbol{h}}{\boldsymbol{w}} \right)}$$

| Total Flow rate | Applied Pressure | Leakage Detection |
| --- | --- | --- |
| (μL/min) | (Bar) |  |
| 100 | 0.05 | No |
| 500 | 0.26 | No |
| 1000 | 0.52 | No |
| 1500 | 0.78 | No |
| 3000 | 1.57 | No |
| 6000 | 3.14 | No |

Table 2S: Summary of pressure testing for microfluidic connectors. Experimental conditions include the test medium loaded in the syringe, applied weight and pressure, and exposure duration for each evaluation.

| Test Medium in Syringe | Load applied | Resultant  Pressure | Exposure Time |
| --- | --- | --- | --- |
|  | (kg) | (Bar) | (Hour) |
| Water | 5.85 | 5 | 3 |
| Air | 1.16 | 1 | 3 |
